# Environmental considerations for post-war reconstruction of Gaza

**DOI:** 10.1007/s13280-025-02246-1

**Published:** 2025-09-23

**Authors:** Naomi L. J. Rintoul-Hynes

**Affiliations:** School of Science, Psychology, Arts and Humanities, Computing, Engineering and Sport, Canterbury Christ Church University, North Holmes Road, Canterbury, Kent, CT1 1QU UK

**Keywords:** Conflict, Ecology, Environment, Habitat, Palestine, Pollution

## Abstract

The environment is often the silent victim of war, but environmental degradation resulting from war is poorly understood. Since the environmental legacy of war can last decades or centuries, environmental peacebuilding and reconstruction efforts must consider the impacts of war on the environment. In the case of the Israel-Palestine conflict, far-reaching and severe environmental damage has been documented. Three key environmental considerations for Gaza are discussed: (I) environmental pollution, (II) habitat degradation and (III) habitat fragmentation. Based on the environmental issues highlighted, recommendations regarding post-war reconstruction efforts are presented.

## Environmental degradation in the context of war

The environment has long been identified as a cause of violent conflict, prompting considerable research on the topic. However, the environment is often the silent victim of war. The term *environcide* describes the process of “damaging, destroying or rendering inaccessible environmental infrastructure through violence” (Kreike 2021, p. 3). Despite this, many warfare-induced environmental issues remain poorly understood, particularly in the context of post-war rebuilding efforts. During conflict, the most pressing horrors of war—injury, death, forced migration, unsanitary living conditions and malnutrition, and so on—take precedent over environmental damage that may be seen as “less dangerous, less painful, and more negligible at the present time” (Kaplan 2022, p. 1514). Thus, these effects to have often been “disregarded, untracked and unaccounted” (Meaza et al. 2024, p. 2). Yet, for post-conflict reconstruction to be successful, there must be a clear understanding of the environmental degradation caused by conflict and the remediation options for improving war-damaged land (Newcombe 2024).

## The case of Gaza

Although the Israeli-Palestinian conflict has been ongoing since 1948, the establishment of the Israeli military blockade since 2007 and numerous flashpoints in fighting have exacerbated environmental issues in the region in recent years. Since the 7th October 2023 attacks by Hamas, the Israeli military offensive saw Palestine become the most violent place in the world (ACLED 2024). So far, 70% of Gaza’s buildings (Dardona et al. 2025), sewage pumps/wastewater treatment plants (Abuawad et al. 2025) and agricultural land (Hassoun et al. 2025) have been damaged or destroyed. Therefore, the war in Gaza has been described as an “environmental catastrophe” (Qumsiyeh 2024, p. 985).

Although the three-phase ceasefire agreement on 19th January 2025 included a final stage focussing on the reconstruction of Gaza. Since this collapsed in 18 March 2025, Israel and Hamas are yet to agree terms on a new ceasefire agreement. However, new proposals (e.g. the United States ‘Witkoff Plan’ proposed in May 2025 and the UN General Assembly Resolution put forward in June 2025) do not include reconstruction commitments. For any ceasefire agreement to be effective long-term, war-related environmental damage must be considered, and rebuilding strategies must include environmental reconstruction. As there is a lack of research examining the environmental impacts of the war in Gaza in the context of post-war reconstruction, this literature review aims to identify key environmental impacts of armed conflict in Gaza and outline relevant recommendations to achieve effective post-war reconstruction.

## Environmental pollution

Conflict can lead to pollution of various environmental compartments (e.g. soil, water and air) that can persist for centuries (Williams and Rintoul-Hynes 2022). Common sources of contamination include the release of components from explosive devices, ammunition, and weapons of mass destruction, oils and lubricants used in military vehicles (Stadler et al. 2022). There is clear evidence that environmental pollution is a common product of these actions (Kaplan et al. 2022), both from historic and current conflict in Gaza (Abuawad et al. 2025). In Gaza, there have been “catastrophic” impacts on infrastructure such as power plants, waste management systems, and water treatment facilities (Shaheen et al. 2024, p. 2). There is also evidence that white phosphorus, a toxic incendiary agent, has been used in Gaza (Qumsiyeh 2024). Other material is left behind in war: it is estimated that 7500 tonnes of non-explosive weapons have been discarded in Gaza (Dardona et al. 2025), in addition to a vast amount of spent ammunition.

As well as pollution from war-related activities, Israel has incentivised polluting industries to build plants near occupied Palestinian territories (Qumsiyeh et al. 2021), exacerbating the problem. In addition, three quarters of water infrastructure has been damaged (Zhao et a., 2024), and water is restricted by blockades (Hassoun et al. 2025). As a result, untreated sewage has been discharged into rivers and seas, exacerbating the problem (Dardona et al. 2025). At one point, at least 130 000 m^3^ of wastewater was discharged into the Mediterranean Sea daily (Shaheen et al. 2024). Large amounts of raw or partially treated sewage are also being released into the Wadi Gaza Nature Reserve (Dardona et al. 2025).

The war has left 37 million tonnes of debris (UNEP 2024)—over 14 times that produced in all global conflicts in the last 16 years (Abuawad et al. 2025). It is thought that it could take “about 14 years of work with 100 trucks" to clear the unexploded ordinance alone (Reuters 2024). The debris also includes 800 000 tonnes of asbestos and other harmful material (Abuawad et al. 2025). In addition, over 1000 tonnes of waste are being produced per day in Gaza—with limited refuse collection, waste is piling up and hazardous substances are being improperly disposed of (UNEP 2024; Dardona et al. 2025).

## Habitat degradation

Palestine accounts for 3% of global biodiversity (Hassoun et al. 2025). This includes 30 904 animal species (Palestinian Central Bureau of Statistics 2016), and 1938 plant species (Ali-Shtayeh and Jamous 2018). Of these, there are 11 critically endangered, 27 endangered, 36 vulnerable and 43 near-threatened species, according to the IUCN Red List (2024). Biodiversity has dramatically declined in recent years, and Hassoun et al. (2025, p. 204) note that “the scale of devastation has disrupted natural ecosystems, obliterated biodiversity, and polluted vital resources like soil and water”. However, the impact of armed conflict on biodiversity, and subsequent conservation requirements, has received little attention (Hilario-Husain et al. 2024).

In Gaza and the West Bank, key habitats include the Wadi Gaza Nature Reserve (Gaza) and Wadi Al-Quff Protected Area (West Bank)—two ecologically important areas of particular scientific interest (Abd Rabou 2019). Wadi Gaza is crucial for migratory birds and the plant communities constitute important habitats for many other species (UNESCO 2012), whereas Wadi Al-Quff has high terrestrial vertebrate biodiversity (Abd Rabou 2019). Many other ecologically important sites have been identified within Gaza and the West Bank (Qumseyih and Abusarhan 2021; Qumseyih et al. 2023).

An obvious cause of habitat degradation is from artillery fire, explosives and landmines used in armed warfare (Lawrence et al. 2015). The effects of these differ: use of explosives to destroy a dam can lead to a sudden substantial loss of species from flooding and siltation downstream or drought upstream, whereas landmines can pose a risk to individual fauna, particularly large mammals (Lawrence et al. 2015). It is likely that tens of thousands of bomb craters and landmines are scattered throughout Gaza (Holail et al. 2024; Qumsiyeh 2024). Similarly, military bases are associated with habitat degradation through land clearance, soil erosion and contamination during their construction and use (Machlis and Hanson 2008; Larence et al. 2015). Similarly, movement of armoured vehicles, military personnel and displaced civilians can lead to similar outcomes (Lawrence et al. 2015; Holail et al. 2024; Qumsiyeh 2024). As a result, deforestation is the leading environmental impact of war (Meaza et al. 2024). Various other activities may harm local habitats. For example, Israeli extraction of sand and groundwater has affected fragile ecosystems (Abd Rabou 2019).

There is evidence of conflict-induced damage to important ecological sites in Gaza, such as the Wadi Gaza Wetlands (Ledur and Chen 2023). Up to half of Wadi Gaza has been destroyed by the current conflict (UNEP 2024). In addition, 90% of Palestinians in Gaza internally displaced (Abuawad et al. 2025), resulting in indirect impacts of the land. Gaza was already one of the most densely populated regions in the world, but now most of the population in concentrated in Al-Mawasi, a coastal strip constituting just 3% of Gaza’s total area (Dardona et al. 2025). As people are displaced, so is wildlife (Hassoun et al. 2025). Half of the region’s agricultural land is destroyed (Sah and Dawas 2024), and Gaza’s livestock has been decimated, losing approximately 99% of poultry, 95% of cattle and half of sheep stocks (Hassoun et al. 2025). Recovery of some sensitive degraded ecosystems in Gaza is expected to take decades (UNEP 2024).

Despite the largely destructive nature of war, it should also be noted that war-related activities can be beneficial for some species. Large, adaptable predators can benefit from changes to the habitat (Lawrence et al. 2015), whereas water-filled bomb craters can provide a habitat for amphibians and insects (Meaza et al. 2024). Military bases can lead to an increase in surface water that attracts a wide range of species, from snakes to migratory birds (Wood, personal communication, March 24, 2024). Finally, land abandonment can allow the land to recuperate, for example through improved vegetation cover due to outmigration of grazers and other species (Lawrence et al. 2015; Meaza et al. 2024). In Gaza, tactical buffer zones have been created, for example at the Netzarim corridor (Boxerman et al. 2024), which may be beneficial to wildlife found there.

## Habitat fragmentation

When parts of a habitat are destroyed in processes described above, it can leave behind smaller unconnected—or *fragmented*—areas. Man-made physical barriers are another key cause of fragmentation in conflict zones. Barriers have been commonplace in Gaza for decades. The initial border fence in Gaza was constructed in 1971, and upgrades have become larger and more complex (Roy 2016). Similarly, Israeli segregation walls have been in Gaza since the 1990s and in the West Bank since 2003 (Husein and Qumsiyeh 2022). These are constructed of barbed wire, electrified fences, concrete walls, trenches or even underground metal walls, with accompanying roads, floodlights and landmines (Trouwborst et al. 2016). Most recently, Israelis constructed the anti-tunnel barrier (the *Iron Wall*) along the entire 40km length of the Gaza-Israel border (Al-Jazeera 2021). Thus, it is unsurprising that even before 2023 the Palestinian Environmental Quality Authority (2015) classed both the West Bank and Gaza at very high risk of environmental degradation from habitat fragmentation.

Firstly, animals can become entangled in razor wire, suffering serious injury or even mortality (Linnell 2016) or can become trapped against electric fences of the Israeli border wall (Abdallah and Swaileh 2011). Structures can impede mobility of animals that are unable to go around them, leading to reduced—or completely ceased—movement of a species (Trouwborst et al. 2016), as is the case in Gaza (Husein and Qumsiyeh 2022). Although the effects are easily imagined for large mammals, barriers can affect smaller species, such as reptiles, insects and birds. This can lead to genetic isolation, and can impact seasonal migrations, nomadic movements and dispersal in search of territories or mates, affecting long-term survival (Trouwborst et al. 2016). Predator animals and human poachers can also take advantage of barriers to trap prey (Trouwborst et al. 2016). In Gaza, these barriers are also known to result in overgrazing pressures, species invasions, changes to hydrology and restrictions on access for conservationists (Husein and Qumsiyeh 2022). Abdallah and Swaileh (2011) also noted the Wall has had impacts on biodiversity. Barriers can also influence vegetation cover, as demonstrated in a study of the West Bank Wall (Meroz et al. 2023), and can act as a barrier to seed dispersal and pollination (Trouwborst et al. 2016).

## Recommendations

Based on the environmental issues described, some key recommendations to achieve sustainable reconstruction of Gaza are given below:

**1**.**Interdisciplinary research on the environmental impacts of warfare should consider all three stages (preparations for war, armed conflict, and post-war activities), as well as short-, medium- and long-term effects.**

Due to obvious risks/limitations during war, most environmental studies are conducted post-war. However, pre-war preparation also leads to increased use of non-renewable resources and environmental degradation (Sidel et al. 2009). Similarly, post-war rebuilding in Gaza will generate annual emissions equivalent to 130 countries (Khan and Bakhsh., 2024). Where possible, pre-war baseline data should be identified to compare to during/post-war data, particularly in regions with notable shortfalls in biodiversity monitoring (Hilario-Husain et al. 2024). Remote sensing can also be utilised to safely monitor environmental conditions before, during, and after conflict (Kahn and Bakhsh 2024).

Some consequences of war are unlikely to be recognised for decades (Abuawad et al. 2025) and restoration efforts often take 10–20 years (Jensen and Lonergan 2012). However, “a detailed, comprehensive, and authoritative study on the actual short-, medium-, and long-term environmental impacts of a single war anywhere in the world is conspicuously absent” (Biswas 2001, p. 308). A rapid environmental assessment should be carried out at the outset of the post-war reconstruction process, followed by comprehensive assessments to assess damage in the medium- to long-term (Jensen and Lonergan 2012). In Gaza, the focus needs to be on assessing how environmental burdens like pollution and water scarcity disproportionately affect the vulnerable population in the strip in the medium- to long-term (Buheji and Al-Muhannadi 2023). Moreover, a holistic approach to land rehabilitation that addresses both environmental and socioeconomic dimensions is key (Hassoun et al. 2025).

**2**.**There must be a transparent, science-based decision-making process that involves all stakeholders (particularly the local community) on environmental restoration.**

It is essential that all stakeholders, including wider civil society, are involved in environmental assessment and decision-making processes as this can build trust between communities, local and national government agencies, and international actors (Jensen and Lonergan 2012). To achieve this, there must be extensive consultations (particularly through workshops and public meetings) at every step of the process and reports must be accessible to the target audience. This will strengthen sense of ownership in the process and increase acceptance of analytical findings and recommendations (Jensen and Lonergan 2012).

To strengthen engagement and ensure long-term sustainable resource management, scientists should work with local communities on projects focussing on conflict-affected areas (Hilario-Husain et al. 2024). In addition, collaborative projects between Palestinian and Israeli scientists should be encouraged (Kahn and Bakhsh 2024). To ensure a fair and transparent process, once all parties agree on a common set of analytical techniques, the responsibility for collection and independent analysis of field samples should be divided between multiple parties (Jensen and Lonergan 2012). Similarly, collaboration with—and funding from—international organisations and the global research community is essential for successful environmental restoration and sustainable development initiatives in Gaza (Hassoun et al. 2025). However, they should take on a supporting role rather than leading efforts, convening workshops, ensure participation of a wide range of stakeholders, sharing best practices and ensuring that the process is run in a fair, open and transparent way (Jensen and Lonergan 2012).

**3**.**Research/policy should consider local and regional habitats, environmental conditions and vulnerabilities.**

Natural resources can drive post-conflict economic growth and stability and can also be used as the basis for regional cooperation and economic integration. Thus, national policies and legislative framework need to support community-based natural resource management and long-term national restoration (Jensen and Lonergan 2012). In Gaza, wetlands, coastal areas and forests play a particularly crucial role in supporting biodiversity; therefore, policies should focus on the restoration and protection of these ecosystems to ensure long-term biodiversity conservation, ecological resilience and environmental sustainability (Hilario-Husain et al. 2024; Hassoun et al. 2025). In addition, rather than focussing solely on habitat degradation, the effects of fragmentation, particularly in small ecological areas, should be considered in planning and policy discussions.

**4**.**A programme to increase awareness of the inherent value of war-affected habitats should be established, involving capacity building, information exchange, awareness programs, educational courses, field work and implementation of environmental legislation.**

Armed conflict may compel local communities to engage in unsustainable coping strategies, such as overharvesting of natural resources (Jensen and Lonergan 2012). However, programmes to understand environmental issues and come up with practical solutions will only work long-term if they run alongside education and awareness campaigns. These should embedded in the school curriculum, disseminated through public workshops, and should involve modern awareness campaigns through social media (Khan and Bakhsh 2024). Practical campaigns should encourage the local community to adopt sustainable practices that mitigate environmental damage while fostering a sense of stewardship (Hassoun et al. 2025). Finally, local capacity building campaigns can lead to the wider local community being trained and directly involved in post-war reconstruction activities.

**5**.**Clearance of war remnants is necessary to ensure safe and clean habitats, not only of unexploded ordinance but of other leftover material such as vehicles, weapons, and ammunition. Hotspots of high contamination risk to ecosystems and/or human health should be identified to target sustainable and cost-effective remediation.**

To identify contamination hotspots, fieldwork should be conducted to analyse environmental samples when appropriate and should enlist expertise of local institutions if possible. Similarly, fieldwork should identify unexploded ordnance or leftover material from war and remove this material where possible. Mine and unexploded ordnance clearance programmes should build local capacity through training and education (Hassoun et al. 2025). To mitigate limitations in data collection, remote sensing and citizen science monitoring schemes can also be utilised to safely monitor the environment before, during, and after conflict (Kahn and Bakhsh 2024; UNEP 2024). When risks are identified (i.e. due to unexploded ordnance or contamination), public awareness campaigns are required to alert the local community to risks, inform them of safe practices, and prevent the spread of misinformation and panic (Jensen and Lonergan 2012).

For polluted areas, common remediation approaches include: (1) isolation (capping or subsurface barriers); (2) immobilisation (solidification/stabilisation, vitrification or chemical treatment); (3) toxicity/mobility reduction (chemical treatment, permeable treatment walls or biological treatment); (4) physical separation; and (5) extraction (soil washing/flushing, pyrometallurgical extraction, and electrokinetic treatment) (Wuana and Okiemen 2011). Key considerations when selecting an approach include the cost, sustainability, ability to reduce/immobilise multiple contaminants, timescale for completion, and local infrastructure/expertise to facilitate the work.

**6**.**Wildlife and biodiversity conservation should be considered during infrastructure (re)development, and environmental impact assessments should be utilised.**

Improvements to infrastructure (i.e. irrigation, water treatment and sewage treatment) are required to ensure sustainable development is achievable; restoring these systems to pre-2023 conditions is not enough. Redevelopment should focus on infrastructure that promotes more sustainable practices (Hassoun et al. 2025). However, it is essential to minimise the environmental impacts of reconstruction itself, particularly on natural resources that are support long-term peacebuilding goals (Jensen and Lonergan 2012). To ensure redevelopment programmes offer long-term environmental sustainability, environmental impact assessments should be used to provide in-depth analysis (Hassoun et al. 2025). In addition, restrictions on the import of materials and technology necessary for environmental restoration (i.e. water purification systems, advanced waste management technologies and building materials) must be lifted. Sustainable redevelopment strategies “are essentially theoretical in the absence of access to necessary resources” (Khan and Bakhsh 2024).

## References

[CR1] Abd Rabou, A. F. N. 2019. The mammalian, reptilian and amphibian fauna of Al-Mawasi Ecosystem, south-western Gaza Strip – Palestine. *Agricultural Research & Technology* 23: 301–314. 10.19080/ARTOAJ.2019.23.556233.

[CR2] Abdallah, T. and K. Swaileh. 2011. Effects of the Israeli segregation wall on biodiversity and environmental sustainable development in the West Bank. *Palestine. International Journal of Environmental Studies* 68: 543–555. 10.1080/00207233.2011.608504.

[CR3] Abuawad, A., M. Griffiths, G. H. Edwards, A. Eftekhari, M. Al-Ebweini, H. Al-Najar, S. Butmeh, R. Abu Dayyeh, et al. 2025. The ongoing environmental destruction and degradation of Gaza: The resulting public health crisis. *American Journal of Public Health* 115: 1053–1061. 10.2105/AJPH.2025.308140. 40499107 10.2105/AJPH.2025.308140 PMC12160645

[CR4] ACLED. 2024. *Conflict Index: December 2024*. https://acleddata.com/conflict-index/

[CR5] Ali-Shtayeh, M. S. and R. M. Jamous. 2018. Updating the plant “red list” of Palestine (West Bank and Gaza strip: Conservation assessment and recommendations. *Journal of Biodiversity and Endangered Species* 6: 2332–2542. 10.4172/2332-2543.1000224.

[CR6] Al-Jazeera. 2021. Israel Completes ‘Iron Wall’ Underground Gaza Barrier. https://www.aljazeera.com/news/2021/12/7/israel-announces-completion-of-underground-gaza-border-barrier.

[CR7] Biswas, A. K. 2001. Scientific assessment of the long-term environmental consequences of war. In *The environmental consequences of war: Legal, economic, and scientific perspectives*, ed. J. E. Austin and C. E. Brush, 303–315. Cambridge: Cambridge University Press.

[CR8] Boxerman, A., A. Toler, R. Mellen, and P. Kingsley. 2024. *Israel Builds Bases in Central Gaza, A Sign It May Be There To Stay*. https://www.nytimes.com/2024/12/02/world/middleeast/israel-gaza-bases-netzarim.html.

[CR9] Buheji, M. and K. Al-Muhannadi. 2023. Mitigating risks of environmental impacts on Gaza—Review of precautions & solutions post (2023 War). *International Journal of Advanced Research in Engineering and Technology* 14: 15–47. 10.17605/OSF.IO/2JMCP.

[CR10] Dardona, Z., M. Amane, A. Dardona, and S. Boussaa. 2025. Health and environmental impacts of Gaza conflict (2023–2024): A review. *One Health Bulletin* 5: 1–12. 10.4103/ohbl.ohbl_42_24.

[CR12] Hassoun, A. H. Jarrar, L. Goudali, S. Lisciani, K. Al-Muhannadi, M. Buheji and A.E.D.A. Bekhit. 2025. Life on Land in Peril: How the Recent War on Gaza Has Devastated Terrestrial Ecosystems. In *War on Gaza*, ed. A. Hassoun, 203–218. Cham: Springer. 10.1007/978-3-031-88500-6_13

[CR13] Hilario-Husain, B. A., K. C. Tanalgo, S. J. C. Guerrero, F. G. N. Garcia, T. E. Lerios, M. A. Z. Garcia, and R. J. Alvaro-Ele. 2024. Caught in the crossfire: biodiversity conservation paradox of sociopolitical conflict. *NPJ Biodiversity* 3: 1–9. 10.1038/s44185-024-00044-8. 39242669 10.1038/s44185-024-00044-8 PMC11332208

[CR14] Holail, S., T. Saleh, X. Xiao, J. Xiao, G. S. Xia, Z. Shao, M. Wang, J. Gong, et al. 2024. Time-series satellite remote sensing reveals gradually increasing war damage in the Gaza Strip. *National Science Review* 11: nwae304. 10.1093/nsr/nwae304. 39309412 10.1093/nsr/nwae304 PMC11413530

[CR01] Husein, D., and M.B. Qumsiyeh, 2022. Impact of Israeli segregation and annexation wall on Palestinian biodiversity. *Africana Studia* 1: 19–26. 10.21747/0874-2375/afr37a2.

[CR15] IUCN. 2024. The IUCN Red List of Threatened Species. Version 2024–2. https://www.iucnredlist.org/.

[CR16] Jensen, D. and S. Lonergan. 2012. Natural resources and post-conflict assessment, remediation, restoration, and reconstruction: Lessons and emerging issues, In *Assessing and restoring natural resources in post-conflict peacebuilding*, eds. D. Jensen and S. Lonergan, 411–462. Abingdon: Earthscan.

[CR17] Kaplan, G., T. Rashid, M. Gasparovic, A. Pietrelli, and V. Ferrara. 2022. Monitoring war-generated environmental security using remote sensing: A review. *Land Degradation & Development* 33: 1511–1771. 10.1002/ldr.4249.

[CR18] Khan, W. A. and K. S. Bakhsh. 2024. Gaza under siege: Environmental disaster during Israel-Palestine conflict, suggestions and solutions. *Margalla Papers* 28: 78–94. 10.54690/margallapapers.28.2.278.

[CR19] Kreike, E. 2021. *Scorched earth*. Princeton, USA: Princeton University Press.

[CR20] Lawrence, M. J., H. L. J. Stemberger, A. J. Zolderdo, D. P. Struthers, and S. J. Cooke. 2015. The effects of modern war and military activities on biodiversity and the environment. *Environmental Reviews* 23: 443–460. 10.1139/er-2015-0039.

[CR21] Ledur, J. and J.K. Chen. 2023. *Tracking Damage Within The Gaza Strip Through Maps*. https://www.washingtonpost.com/world/2023/gaza-strip-map-attacks-damage/.

[CR22] Linnell, J. 2016. Refugee fences fragment wildlife. *Nature* 529: 156. 10.1038/529156a. 10.1038/529156a 26762448

[CR23] Machlis, G. E. and T. Hanson. 2008. Warfare ecology. *BioScience* 58: 729–736. 10.1641/B580809.

[CR24] Meaza, H., T. Ghebreyohannes, J. Nyssen, Z. Tesfamariam, B. Demissie, J. Poesen, M. Gebrehiwot. 2024. Managing the environmental impacts of war: What can be learned from conflict-vulnerable communities? *Science of the Total Environment* 927: 171974. 10.1016/j.scitotenv.2024.171974. 38547990 10.1016/j.scitotenv.2024.171974

[CR02] Meroz, A.M., H. Yin, and N. Levin, 2023. Unveiling the impact of traditional land practices on natural vegetation using large-scale exclosures: National borders and military bases. *Journal of Arid Environments* 211: 104930. 10.1016/j.jaridenv.2023.104930.

[CR25] Newcombe, P. B. 2024. More studies are needed on the long-term environmental consequences of war. *Nature* 632: 739. 10.1038/d41586-024-02711-z. 10.1038/d41586-024-02711-z 39164567

[CR26] Palestinian Central Bureau of Statistics. 2016. *Press Release by Palestinian Central Bureau of Statistics (PCBS) and the Environment Quality Authority on World Environment Day*. https://www.pcbs.gov.ps/post.aspx?lang=en&ItemID=1678.

[CR27] Qumsiyeh, M. B. 2024. Impact of the Israeli military activities on the environment. *International Journal of Environmental Studies* 81: 977–992. 10.1080/00207233.2024.2323365.

[CR28] Qumsiyeh, M. B., D. Hussein, N. Boulad, I. M. Albaradeiya, M. Mahasnah, M. Abusarhan, M. Najajrah, B. Al-Shaikh, et al. 2023. Updating and enhancing the Protected Areas Network of Palestine: A step towards biodiversity conservation. *Parks* 29: 73–84. 10.2305/UBEA6691.

[CR29] Qumsiyeh, M.B. and M.A. Abusarhan. 2021. Biodiversity and Environmental Conservation in Palestine. In *Biodiversity, conservation and sustainability in Asia*, eds. M. Öztürk, V. Altay, and R. Efe, 1–22. Cham: Springer, 10.1007/978-3-030-59928-7_1.

[CR30] Reuters. 2024. *UN Official Says It Could Take 14 Years to Clear Debris in Gaza*. https://www.reuters.com/world/middle-east/un-official-says-it-could-take-14-years-clear-debris-gaza-2024-04-26/.

[CR31] Roy, S.M. 2016. *The Gaza Strip: The Political Economy of De-Development*. Beirut, Lebanon: Institute for Palestine Studies.

[CR32] Sah, S. and K. Dawas. 2024. Israel is using starvation as a weapon of war in Gaza. *BMJ* 385: q1018. 10.1136/bmj.q1018. 38719494 10.1136/bmj.q1018

[CR33] Shaheen, A., R. Dajani, K. Zinszer, Y. Ashour, and S. Abuzerr. 2024. The war on the Gaza Strip and its consequences on global warming. *Frontiers in Human Dynamics* 6: 1463902. 10.3389/fhumd.2024.1463902.

[CR34] Sidel, V.W., B.S. Levy and J.E. Slutzman. 2009. Prevention of war and its environmental consequences. In *Environmental consequences of war and aftermath*, eds. T.A. Kassim and D. Barceló, 21–39. Berlin: Springer. 10.1007/978-3-540-87963-3_2.

[CR35] Stadler, T., Á. Temesi, and Z. Lakner. 2022. Soil chemical pollution and military actions: A bibliometric analysis. *Sustainability* 14: 7138. 10.3390/su14127138.

[CR36] Trouwborst, A., F. Fleurke, and J. Dubrulle. 2016. Border fences and their impacts on large carnivores, large herbivores and biodiversity: An international wildlife law perspective. *Review of European Community & International Environmental Law* 25: 291–306. 10.1111/reel.12169.

[CR37] UNESCO. 2012. *Wadi Gaza Coastal Wetlands (Tentative List 5722)*. https://whc.unesco.org/en/tentativelists/5722/.

[CR38] United Nations Environment Programme (UNEP). 2024. *Environmental Impact of the Conflict in Gaza Preliminary Assessment of Environmental Impacts.* Nairobi, Kenya: United Nations Environment Programme.

[CR39] Williams, O. H. and N. L. J. Rintoul-Hynes. 2022. Legacy of war: Pedogenesis divergence and heavy metal contamination on the WWI front line a century after battle. *European Journal of Soil Science* 73: 13297. 10.1111/ejss.13297.

[CR40] Wood, M. 2024. Conversation with Martin Wood, March 24, 2024

[CR41] Wuana, R. A. and F. E. Okiemen. 2011. Heavy metals in contaminated soils: A Review of sources, chemistry, risks and best available strategies for remediation. *ISRN Ecology* 2011: 1–20. 10.5402/2011/402647.

[CR42] Zhao, Q., X. Tan, Q. Meng, L. Gao, L. Zhang, X. Lei, W. Shi, and M. Zhu. 2024. Satellite and AI monitoring humanitarian crises in the Gaza Strip during the early stage of Israeli-Palestinian conflict. *International Journal of Digital Earth* 17: 2430678. 10.1080/17538947.2024.2430678.

